# Integration of Continuous Glucose Monitoring With HbA1c to Improve the Detection of Prediabetes in Asian Individuals: Model Development Study

**DOI:** 10.2196/81520

**Published:** 2026-04-27

**Authors:** Michelle H Lee, Shihui Jin, Eveline Febriana, Maybritte Lim, Sonia Baig, Shahmir H Ali, Ian Yi Han Ang, Tze Ping Loh, Ashna Nastar, Kee Seng Chia, Alice Pik-Shan Kong, Faidon Magkos, Alex R Cook, Sue-Anne Toh

**Affiliations:** 1 Department of Medicine, Yong Loo Lin School of Medicine, National University of Singapore and National University Health System, 10 Medical Drive, Singapore, 117597, Singapore, 65 90012612; 2 NOVI Health, Singapore, Singapore; 3 Saw Swee Hock School of Public Health, National University of Singapore and National University Health System, Singapore, Singapore; 4 Department of Clinical and Translational Research Office, National Heart Centre, Singapore, Singapore; 5 Department of Radiation Oncology, National University Cancer Institute, Singapore, Singapore; 6 Department of Laboratory Medicine, National University Hospital, Singapore, Singapore; 7 Department of Medicine, Alexandra Hospital, Singapore, Singapore; 8 Department of Medicine and Therapeutics, Faculty of Medicine, Chinese University of Hong Kong, Hong Kong, China (Hong Kong); 9 Department of Nutrition, Exercise and Sports, University of Copenhagen, Copenhagen, Denmark; 10 Department of Medicine, National University Hospital, National University Health System, Singapore, Singapore

**Keywords:** continuous glucose monitoring, HbA_1c_, machine learning, screening, Asian, prediabetes, glycated hemoglobin

## Abstract

**Background:**

Glycated hemoglobin (HbA_1c_) is a convenient tool to evaluate glycemic status but its ability to detect individuals at risk for type 2 diabetes is limited.

**Objective:**

Exploiting the glycemic variability captured in continuous glucose monitoring (CGM), we used a well-characterized Asian cohort study from Singapore to assess whether utilizing CGM features in a machine learning model can improve the detection of prediabetes as compared to using HbA_1c_ alone.

**Methods:**

In this study, 406 nondiabetic Asian participants underwent an oral glucose tolerance test and had their fasting and 2-hour plasma glucose concentrations measured, together with HbA_1c_, to classify them as with normoglycemia or prediabetes. They also wore a CGM sensor for 14 days. CGM profile features were extracted and prediction models were constructed with random subsampling validation to evaluate predictive efficacy. The use of CGM and HbA_1c_ data alone or in combination was assessed for the ability to correctly distinguish prediabetes from normoglycemia.

**Results:**

In this cohort (N=406), 189 (46.6%) individuals had prediabetes. The majority of the cohort were women (n=236, 58.1%) and of Chinese ethnicity (n=267, 65.8%). Those with prediabetes were slightly older, heavier, and had higher glucose levels with more variability than the normoglycemia group. A 2-step approach was used where those with HbA_1c_ ≥5.7% were automatically categorized as having prediabetes; the model then focused on the prediction capability of the CGM features among individuals with HbA_1c_ <5.7%. The prediction models with CGM outperformed the benchmark for comparison defined by HbA_1c_ ≥5.7%, where they yielded an area under the receiver operating characteristic curve of 0.866‐0.876, with a lower specificity of 78%‐80% but a vastly improved sensitivity of 76%‐78%.

**Conclusions:**

Adding CGM to HbA_1c_ in a 2-step approach greatly improved the sensitivity of detecting prediabetes in an Asian population. Given the benefits to optimizing lifestyle behaviors and its growing acceptability among the nondiabetic population, CGM is a promising alternative for type 2 diabetes mellitus risk screening.

## Introduction

Glycated hemoglobin (HbA_1c_) is the gold standard test for blood glucose monitoring and the management of diabetes in clinical practice, after 2 landmark prospective clinical studies (US Diabetes Control and Complications Trial [1] and the UK Prospective Diabetes Study [2]) clearly demonstrated a link between HbA_1c_ values and diabetes-related long-term complications, underpinning the use of HbA_1c_ as a surrogate marker for diabetes outcomes [3]. In 2010, HbA_1c_ was incorporated into the American Diabetes Association (ADA) criteria as a screening tool for prediabetes [4]. The advantages of HbA_1c_ include the absence of the requirement for fasting or timed blood samples, and its relative stability compared with glucose concentrations of fasting and 2 hours after an oral glucose tolerance test (OGTT), thereby reflecting long-term (~3 months) glycemic status. However, HbA_1c_ has a limited sensitivity to detect individuals at high risk of type 2 diabetes mellitus (T2DM) [5]. Data from 5395 nondiabetic individuals from the National Health and Nutrition Examination Survey showed that the current 5.7% cut-off has low sensitivity in detecting prediabetes, implying that HbA_1c_ values below 5.7% do not reliably exclude the presence of prediabetes [6]. Moreover, HbA_1c_ can be affected by blood disorders such as anemia [7], but also ethnicity [8], and does not provide any information on glycemic variability (ie, fluctuations in glucose levels over the course of a day).

The continuous glucose monitoring (CGM) system measures glucose levels subcutaneously in the interstitial fluid at regular time intervals for about 1‐2 weeks. Since the first version by Medtronic in 1999, CGM has revolutionized glucose management in diabetes where excursions in glucose can be detected almost instantaneously, providing real-time information to better optimize medications for glucose control [9]. With the improvements in CGM technology, ease of use, and possible integration with other wearable biosensors, CGM is no longer confined to diabetes management. It is attracting the attention of healthy, nondiabetic individuals who want to assess their risk for T2DM or optimize their health [10]. Advances in data science and the application of machine learning techniques [11] have made it possible to exploit the glycemic variability captured in the CGM data to identify distinct patterns of glucose dysregulation for targeted clinical action [12,13], as well as distinguish those who are at risk of T2DM from those who are not [14-16].

Population-based screening can identify those who are at risk of T2DM (ie, prediabetes) to allow for early intervention and thereby mitigate the growing burden of the diabetes epidemic. This is critically important as almost one-in-two adults (45% or 240 million globally) living with diabetes (20‐79 years old) are unaware of their status [17]. Recommended criteria for diagnosing prediabetes and T2DM encompass cut-offs for HbA_1c_, but also fasting and 2-hour OGTT glucose concentrations [18], even though the OGTT is tedious and time-consuming, thereby reducing the effectiveness of a population-wide screening program. However, using HbA_1c_ alone is not sensitive enough to capture individuals at risk. There have been recent reports utilizing CGM data as a potential screening tool to discriminate between healthy individuals and those at risk of T2DM in European, Caucasian [14,16] and Indian [15] populations, but studies are scarce, especially in Asia, where 60% of all diabetes reside [19]. In this study, we took advantage of a well-characterized Asian cohort study from Singapore to assess whether utilizing CGM features in a machine learning model can improve the identification of prediabetes compared with HbA_1c_ alone.

## Methods

### Study Participants

This is a substudy anchored within the “Assessing the Progression to Type-2 Diabetes” (APT-2D) study (ClinicalTrials.gov: NCT02838693), which follows up on a large cohort of nondiabetic individuals for 3 years or until they develop T2DM [20].

For the main APT-2D study, healthy participants between the ages 30 and 70 years old, who were not on any long-term medication and had no prior history of diabetes, were recruited from April 2016 to December 2018. Recruitment was done through random sampling via various outreach efforts to the grassroots communities and organizations, and through media releases in order to recruit at least 2300 participants, so as to ensure adequate sample size for the cohort study outcomes.

For this substudy, participants with their penultimate visit (75 g OGTT) being conducted within 3 months from enrolling to this substudy (“Continuous Glucose Monitoring to Assess Glucose Dysregulation in Progression to Type-2 Diabetes” [CGM-APT2D]) and willing to wear a CGM sensor for 14 days were invited to participate. Out of the 449 individuals enrolled in this substudy, 429 participants contributed with CGM data. To narrow the study’s focus to the Asian population in this region, individuals from Europe or the Middle East were excluded from the analysis. Additionally, those diagnosed with T2DM during the study were removed to align with the study’s objective of examining individuals without diabetes. At the end, data from 419 individuals were used.

### Ethical Considerations

The main study (APT-2D) was approved by the Domain Specific Review Board of the National Health Group (ref: 2016/00096) in Singapore. This substudy (CGM-APT2D) was approved by the Domain Specific Review Board of the National Healthcare Group (ref: 2020/01085) in Singapore. All participants provided written informed consent prior to joining the studies.

### Experimental Procedures

#### OGTT

Participants were admitted after having fasted overnight for 10‐12 hours. They were instructed to abstain from performing any strenuous exercise during the previous day and from consuming fat-rich foods during the preceding 3 days, to avoid potential delayed metabolic effects of exercise and high-fat feeding on metabolism. Participants arrived in the morning (8 AM); height, weight, and vital signs (heart rate and blood pressure) were obtained by standard methods after 5 minutes of rest, before any testing began. Participants then underwent a 2-hour OGTT. An indwelling catheter was inserted into an antecubital vein of one arm for blood sampling. A fasting blood sample was obtained at *t*=0 minutes, and then participants ingested a solution containing 75 g of glucose; additional blood samples were obtained at *t*=10, 20, 30, 60, 90 and 120 minutes for measurement of glucose concentrations. HbA_1c_ was measured using the fasting blood sample. Prediabetes and T2DM were defined by using the ADA criteria [18] for fasting and 2-hour plasma glucose and HbA_1c_ as previously described [20]. Participants classified as having newly diagnosed diabetes were excluded from the analysis.

#### CGM

A CGM sensor (Abbott Freestyle Libre) was attached to the upper arm to monitor interstitial glucose levels at 15-minute intervals for 14 days. The Freestyle Librelink app (Abbott) was used on participants’ smartphones to scan and record their glucose readings. If a participant did not have a compatible smartphone, a reader was loaned to the participant to record glucose readings. Participants were instructed to scan the sensor at least once every 8 hours and to keep the sensor on for 14 days. Participants who had their sensors detached were offered a replacement sensor. The CGM sensor was worn by the participants within 3 months after completing the OGTT visit.

### Sample Analysis

HbA_1c_ was measured in whole blood by cation-exchange high performance liquid chromatography (Bio-Rad Variant II Turbo; Bio-Rad). Plasma glucose during the OGTT was determined by the AU5822 general chemistry analyzer (Beckman Coulter) at the National University Hospital Referral Laboratory (accredited by the College of American Pathologists).

### Data and Statistical Analyses

For each participant, the data encompassed 3 main components: demographic information, including age, sex, and ethnicity; clinical measurements recorded during the clinic visit, consisting of BMI, waist-hip ratio, fasting and 2-hour OGTT glucose (mmol/L), and HbA_1c_ (%); and glucose profile data for about 14 days obtained from the worn CGM sensor. The CGM data comprised a time series capturing the time and corresponding glucose levels (mmol/L), spaced at approximately 15-minute intervals. These intervals are not strictly equal, and the recording length and timing varied among individuals. Participants scanned the CGM sensor at an average frequency of 9.1 times a day. There were additional heterogeneities in the CGM datasets. For instance, occasional involuntary detachment of the sensor resulted in missing data, while the malfunctioning of sensors in some participants resulted in them wearing more than 1 sensor during the study period. Of the 429 participants, 8% required 1 replacement sensor and 0.5% required 2 replacement sensors.

For ease of comparison, we followed the work by Mao et al [13] and converted the CGM measurements into meaningful features that capture the essential characteristics of an individual’s glucose trajectory. These features encompass measures of centrality (mean) and spread (maximum, minimum, standard deviation, coefficient of variation, and mean amplitude of glycemic excursion), along with proportions of time spent within abnormal ( >7.8 or <3 mmol/L) and normal (3 to 7.8 mmol/L) glucose ranges. We additionally considered measures of glucose excursion, including the average rise and fall and their corresponding rates. Further details about the various CGM features, in terms of how they were defined and derived, are provided in Multimedia Appendix 1.

These summary statistics were computed after excluding the first 24 hours in each consecutive block of CGM recordings, as recommended by the manufacturer because glucose readings on the first day were typically lower compared to subsequent days. In cases where participants wore more than 1 device, we merged CGM recordings from different blocks after eliminating data from the “warm-up” period, given the negligible time gaps between them. During this process, 11 participants were subsequently removed due to insufficient CGM data captured by their devices, preventing the calculation of meaningful CGM summary statistics. Two participants were removed due to invalid HbA_1c_ values. Consequently, the final dataset comprises information from 406 participants with an average CGM recording length of 12 days.

We categorized these 406 participants based on their diabetic status into those with prediabetes and those with normoglycemia and prediabetes, thereby enabling a comparative analysis between groups. Specifically, for categorical variables such as sex and ethnicity, we used the Pearson chi-square test, while for continuous variables, we explored potential differences in group means through nonparametric Mann-Whitney *U* tests. *P* values were computed and a significance level of .05 was applied for interpretation.

Prediction models were then constructed to assess individual prediabetes risk. We explored three distinct sets of predictors in our analysis (Table S1 in Multimedia Appendix 1):

1. Demographic data encompassing age and gender, and 2 fundamental clinical measurements, namely BMI and waist-hip ratios (henceforth referred to as “Demo”);
2. A combination of all the CGM summary statistics and the Demo data specified in set 1 (“CGM”);
3. The CGM dataset listed in set 2, supplemented with the numerical HbA_1c_ value (“HbA_1c_”).

The performances of these models were compared with HbA_1c_ ≥5.7% alone to classify prediabetes as the benchmark for comparison.

For each of these data configurations, we employed 2 classification algorithms: logistic regression (LR) and support vector machine (SVM). To address potential concerns of overfitting, we implemented repeated random subsampling validation strategies, and evaluated the average prediction performance across 1000 randomly selected test sets, utilizing key metrics such as misclassification rates, specificity, and sensitivity.

**Figure 1.**
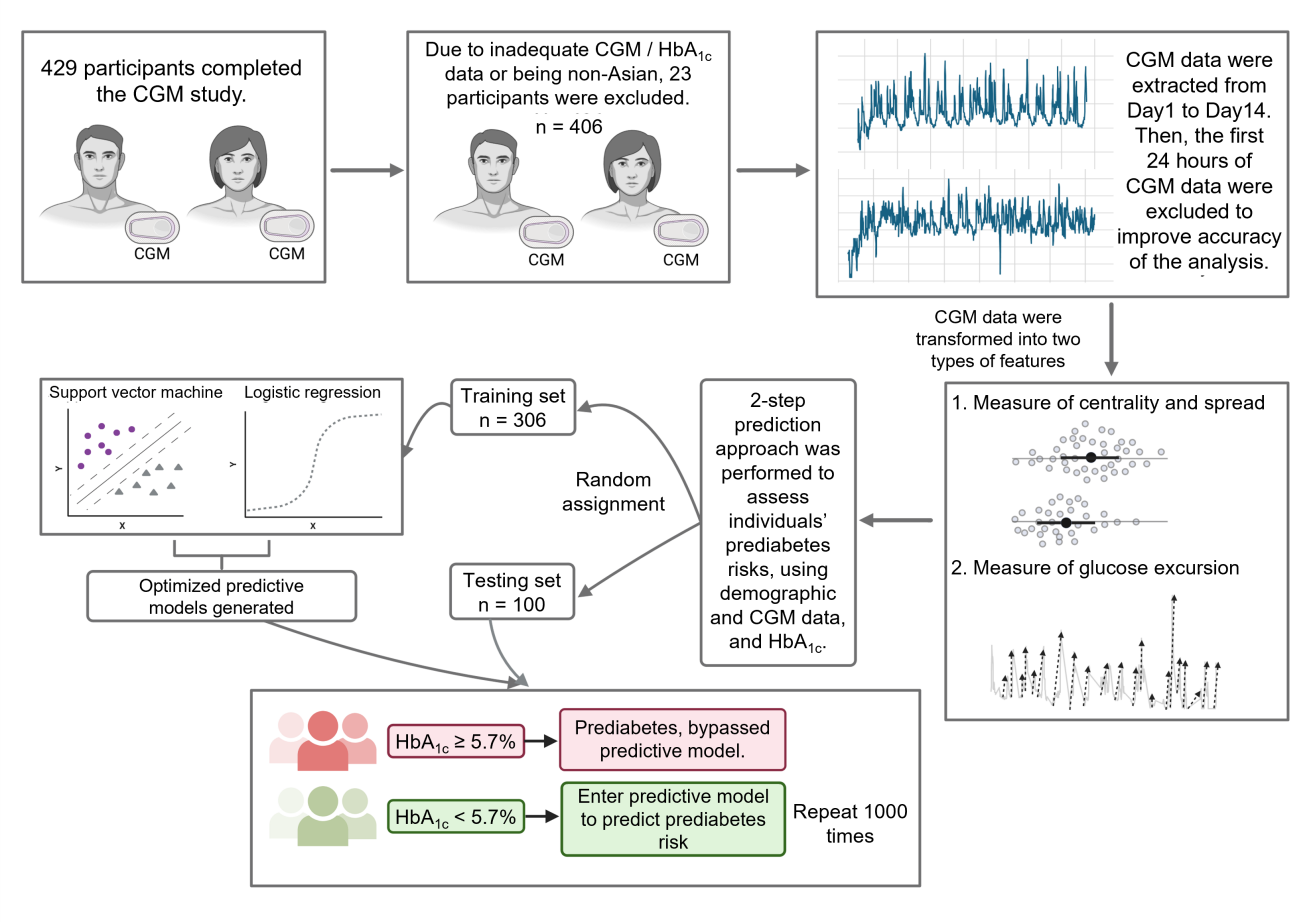
Graphical overview of methods and analysis of CGM data. CGM: continuous glucose monitoring; HbA_1c_: glycated hemoglobin.

In addition to our primary prediction models, we introduced a specialized 2-step prediction strategy. This approach categorized individuals with HbA_1c_ ≥5.7% as having prediabetes and focused exclusively on predicting the prediabetes risk among individuals with HbA_1c_ <5.7%. The predictor sets employed for this targeted analysis included the CGM dataset (set 2) and the HbA_1c_ dataset (set 3). We explored whether this segregation would enhance risk prediction process, allowing for a more nuanced understanding of prediabetes risk among individuals with relatively low HbA_1c_ levels. A sensitivity analysis was additionally conducted regarding the segregation threshold of 5.7%, but the alternative thresholds failed to yield better prediction accuracy in terms of misclassification rates (Tables S2–S3 in Multimedia Appendix 1). An overview of the methods is summarized in Figure 1.

## Results

### Participant Demographics and Baseline Clinical and CGM Measurements

Demographic information and clinical glucose measurements of the 406 Asian participants without diabetes are summarized in Table 1. Within this cohort, 189 (46.6%) individuals were categorized as prediabetic. The majority of the cohort were women (n=236, 58.1%) and individuals of Chinese ethnicity (n=267, 65.8%), with no significant differences observed in terms of sex and ethnic group distribution between the normoglycemia and prediabetes groups. The age of participants spanned from 33 to 74 years, with half falling between 42 and 57 years old. Those in the prediabetes group were ~6 years older and had a slightly greater BMI and waist-to-hip ratio than those in the normoglycemia group; the prevalence of obesity was 32.3% and 19.8% respectively, under the Singapore classification system [21]. Fasting and 2-hour OGTT glucose levels and HbA_1c_ were significantly greater in prediabetic than normoglycemic individuals (Table 1 and Figure S1 in Multimedia Appendix 1).

**Table 1.** Demographics and baseline clinical glucose measurements in Asians without diabetes. Comparison between normoglycemia and prediabetes groups was performed using the Pearson chi-square test for categorical variables (sex and ethnicity), and the Mann-Whitney *U* test for continuous variables (ie, all but sex and ethnicity).

Variable	Total (N=406)	Normoglycemia (n=217)	Prediabetes (n=189)	*P* value
Sex, n (%)				.40
Male	170 (41.9)	95 (43.8)	75 (39.7)	
Female	236 (58.1)	122 (56.2)	114 (60.3)	
Ethnicity, n (%)				.46
Chinese	267 (65.8)	143 (65.9)	124 (65.6)	
Malay	57 (14.0)	27 (12.4)	30 (15.9)	
Indian	63 (15.5)	34 (15.7)	29 (15.3)	
Others	19 (4.7)	13 (6.0)	6 (3.2)	
Age (years), median (IQR)	49 (42-57)	46 (40-55)	52 (46-60)	<.001
BMI (kg/m^2^), median (IQR)	24.7 (21.9-27.6)	24.0 (22.0-27.0)	25.0 (22.0-29.0)	.01
Waist-to-hip ratio, median (IQR)	0.87 (0.82-0.91)	0.86 (0.81-0.90)	0.88 (0.83-0.92)	.009
Fasting glucose (mmol/L), median (IQR)	5.0 (4.6-5.2)	4.8 (4.6, 5.1)	5.2 (4.8-5.4)	<.001
2-hour OGTT^a^ glucose (mmol/L), median (IQR)	6.9 (5.8-8.2)	6.1 (5.2-6.9)	8.3 (7.2-9.3)	<.001
HbA_1c_^b^ (%), median (IQR)	5.5 (5.3-5.7)	5.3 (5.2-5.5)	5.7 (5.5-5.9)	<.001

a OGTT: oral glucose tolerance test.

b HbA_1c_: glycated hemoglobin.

**Figure 2.**
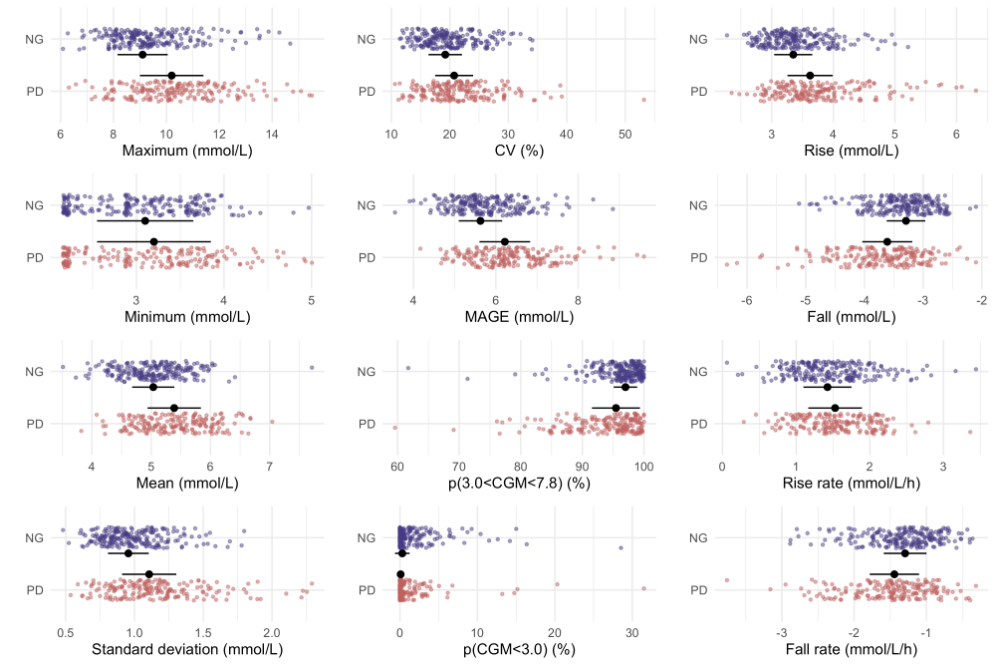
Continuous glucose monitoring (CGM) glucose metrics in Asians with normoglycemia (NG) and prediabetes (PD). Significant differences were observed in all metrics except for the minimum glucose. CV: coefficient of variation; MAGE: mean amplitude of glycemic excursion.

With respect to glucose features derived from the CGM data, the prediabetes group displayed larger variability in glucose levels over time compared to the normoglycemia group, substantiated by significant differences in group means for most measurements related to spread and glucose excursion; only the minimum glucose was not significantly different between the 2 groups (Figure 2 and Table S4 in Multimedia Appendix 1). Notably, our assessment of time-in-range focused on the interval of 3.0‐7.8 mmol/L. This choice stemmed from the consideration that the conventional cut-off for time-in-range (3.9‐10 mmol/L) did not appear pertinent in this nondiabetic population, given the minimal divergence in the proportions of time spent outside this interval between the 2 groups (Table S5 in Multimedia Appendix 1).

### Feasibility of Diagnosing Prediabetes Utilizing CGM Features

**Figure 3.**
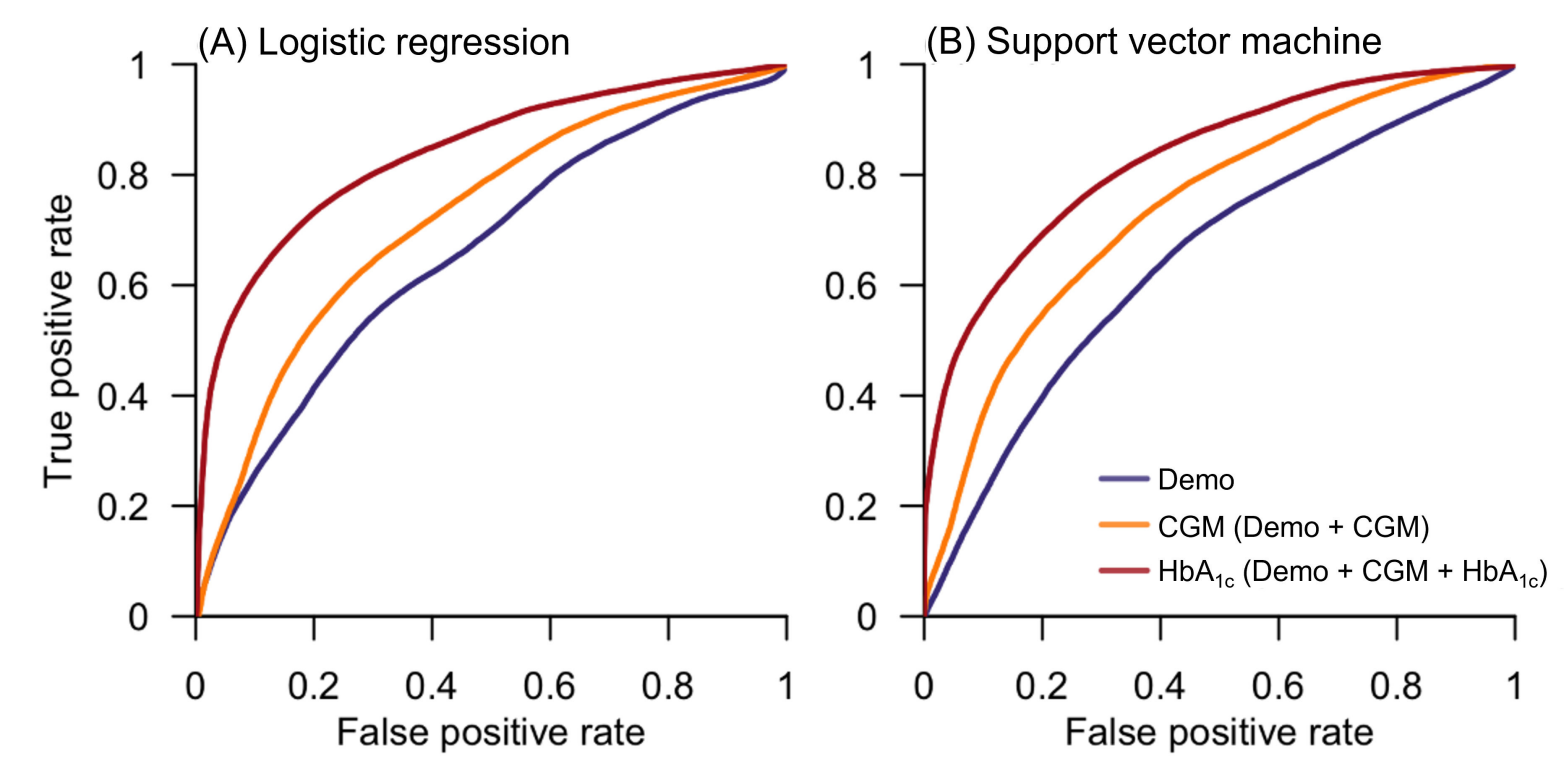
Receiver operating characteristic curves of the 3 model variants utilizing (A) logistic regression or (B) support vector machine as the classification algorithm. Model Demo: age, gender, BMI, and waist-hip ratio; Model CGM: Demo + CGM features; Model HbA_1c_: Demo + CGM + HbA_1c_. CGM: continuous glucose monitoring; HbA_1c_: glycated hemoglobin.

We set the benchmark for comparison using the prediabetes cut-off defined by HbA_1c_ ≥5.7%, resulting in a specificity of 100%, sensitivity of 60%, and a misclassification rate of 18%. The Demo model, which incorporated basic demographic information and obesity-related measures (age, gender, BMI, and waist-to-hip ratio) that are known risk factors for T2DM (Table S1 in Multimedia Appendix 1), demonstrated limited efficacy in distinguishing prediabetes from normoglycemia (Figure 3 and Table 2). The predictive performance greatly improved with the inclusion of CGM features in the model (CGM model), with a prediction sensitivity of 60%‐63% becoming comparable to that using HbA_1c_ ≥5.7% (Table 2). The diagnostic capability further increased when the HbA_1c_ value was added as a predictor to the CGM model (HbA_1c_ model), yielding a higher specificity of 78%‐80% and a sensitivity of 71%‐74%, and hence a substantially lower misclassification rate of 23%‐25% (Table 2). Notably, the sensitivity achieved by the HbA_1c_ prediction model surpassed that obtained when using HbA_1c_ ≥5.7% as a single threshold of 60%. In addition, the choice of classification algorithms did not fundamentally impact the predictive performance for all 3 models (Figure 3 and Table 2).

**Table 2.** Prediction accuracy of prediabetes, in terms of misclassification rates, specificity, and sensitivity, for model variants using distinct predictor sets and classification algorithms. The data are presented as the mean and 95% confidence intervals, derived from 1000 random splits into training and testing sets.

Model	Misclassification (%)	Specificity (%)	Sensitivity (%)	ROC AUC^b^
Demo, mean (95% CI)				
LR^c^	37.1 (36.8-37.4)	70.7 (70.4-71.1)	53.7 (53.3-54.2)	0.658 (0.654-0.661)
SVM^d^	38.0 (37.7-38.3)	71.6 (71.3-72.0)	50.9 (50.4-51.3)	0.648 (0.644-0.651)
CGM^e^, mean (95% CI)				
LR	32.3 (32.0-32.6)	74.4 (74.1-74.8)	59.9 (59.5-60.3)	0.723 (0.719-0.726)
SVM	31.9 (31.6-32.2)	72.7 (72.3-73.1)	62.8 (62.4-63.3)	0.740 (0.736-0.743)
HbA_1c_^f^, mean (95% CI)				
LR	23.2 (23.0-23.5)	79.5 (79.1-79.8)	73.6 (73.2-74.0)	0.838 (0.836-0.841)
SVM	25.2 (24.9-25.4)	77.9 (77.5-78.2)	71.3 (70.9-71.7)	0.829 (0.826-0.831)
HbA_1c_ ≥5.7% cut-off (benchmark)	18.5	100	60.3	0.802

a Model Demo: age, gender, BMI and waist-hip ratio; Model CGM: Demo + CGM features; Model HbA_1c_: Demo + CGM + HbA_1c_.

b ROC AUC: area under the receiver operating characteristic curve.

c LR: logistic regression.

d SVM: support vector machine.

e CGM: continuous glucose monitoring.

f HbA_1c_: glycated hemoglobin.

### Enhancing Prediction Efficacy Through the 2-Step Approach

**Figure 4.**
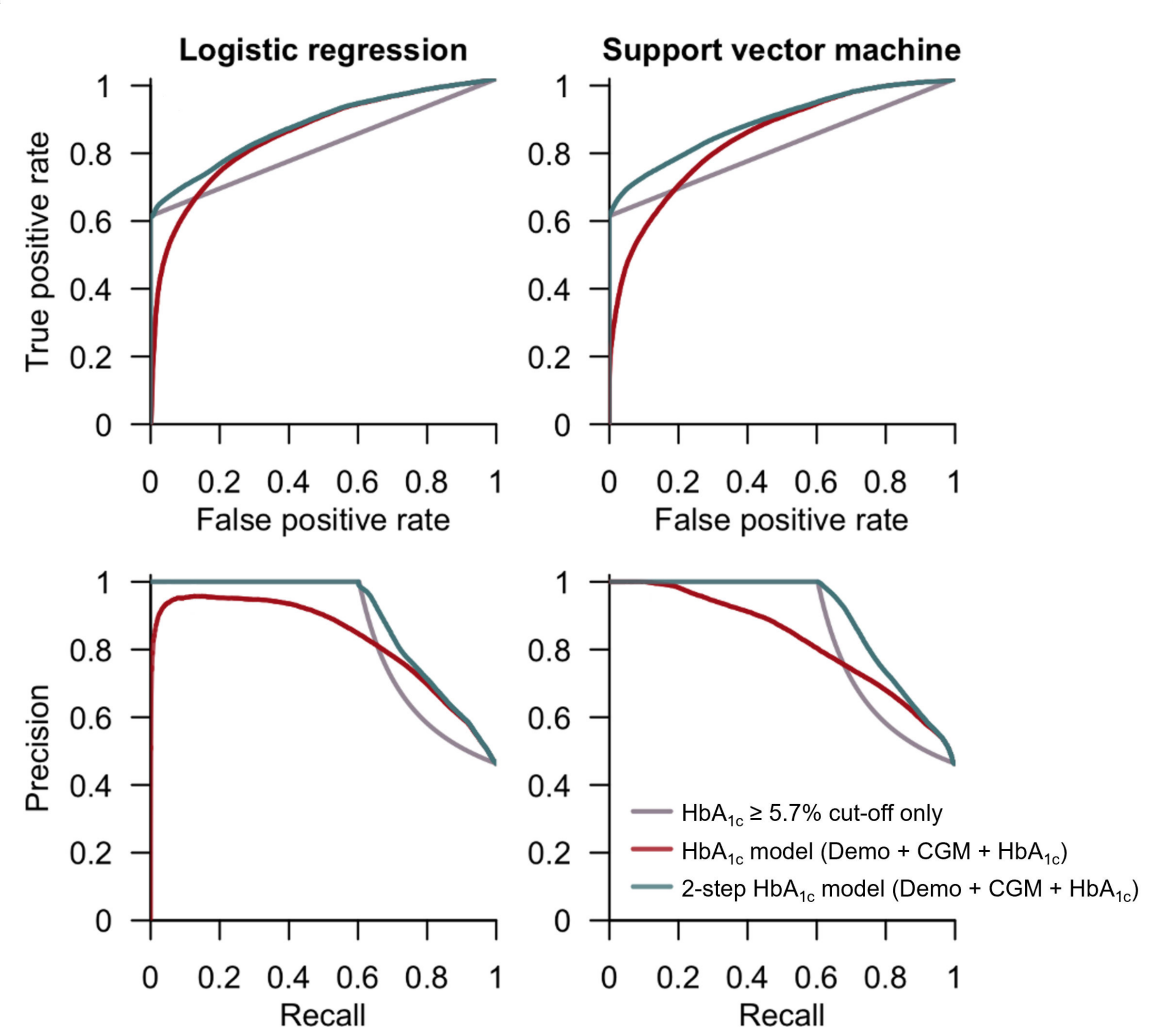
The receiver operating characteristic and precision recall curves comparing the original model HbA_1c_ with the 2-step approach utilizing logistic regression or support vector machine as the classification algorithm. HbA_1c_ ≥5.7% cut-off to classify prediabetes was used as the benchmark for comparison. CGM: continuous glucose monitoring; HbA_1c_: glycated hemoglobin.

We extended our analysis to assess whether stratifying the population based on their HbA_1c_ levels could improve the detection of prediabetes using a 2-step approach where those with HbA_1c_ ≥5.7% were automatically categorized as having prediabetes, thereby focusing the prediction capability on those with HbA_1c_ <5.7%. Overall, this 2-step approach outperformed the benchmark for comparison using the prediabetes cut-off defined by HbA_1c_ ≥5.7%, and significantly improved the predictive performance in the HbA_1c_ model of both algorithms where LR_ROC AUC_ increased from 0.838 to 0.866 and SVM_ROC AUC_ increased from 0.829 to 0.876 (Tables2  3 and Figure 4). In the LR HbA_1c_ model, there was an increase in sensitivity from 73.6% to 75.7%, resulting in a net reduction of the misclassification rate from 23.2% to 22.3%. A similar trend was also observed when SVM was used (Tables2  3). Interestingly, the inclusion of the HbA_1c_ value as a variable in the 2-step approach did not improve the predictive performance in both the LR (area under the receiver operating characteristic curve [ROC AUC] of CGM: 0.872 vs ROC AUC of HbA_1c_: 0.866) and SVM (ROC AUC of CGM: 0.881 vs ROC AUC of HbA_1c_: 0.876) models (Table 3). Assessment of predictive performance using the area under the precision recall curve yielded a similar outcome (Table 3). Nonlinear models such as random forest and Extreme Gradient Boosting were also explored (Table S6 in Multimedia Appendix 1) but were not superior in predictive performance compared to the LR and SVM models in the 2-step approach (Table S7 in Multimedia Appendix 1 vs Table 3). Thus, LR and SVM were chosen for their ease of interpretability and clinical usability.

**Table 3.** Prediction accuracy of prediabetes, including misclassification rate, specificity, and sensitivity, for all data points within the testing sets. This evaluation was conducted for model variants that used the 2-step prediction strategy and drew inference from all data points in the training sets, irrespective of their glycated hemoglobin (HbA_1c_) levels. The data are presented as the mean and 95% confidence intervals, derived from 1000 random splits into training and testing sets.

Model^a^	Misclassification (%)	Specificity (%)	Sensitivity (%)	ROC AUC^b^	PRC AUC^c^
CGM^d^, mean (95% CI)					
LR^e^	22.5 (22.2-22.7)	74.4 (74.1-74.8)	81.1 (80.7-81.4)	0.872 (0.869-0.874)	0.893 (0.891-0.895)
SVM^f^	23.1 (22.8-23.4)	72.7 (72.3-73.1)	81.8 (81.4-82.2)	0.881 (0.879-0.883)	0.899 (0.897-0.901)
HbA_1c_, mean (95% CI)					
LR	22.3 (22.0-22.5)	79.5 (79.1-79.8)	75.7 (75.3-76.1)	0.866 (0.863-0.868)	0.889 (0.887-0.891)
SVM	21.9 (21.6-22.1)	77.9 (77.5-78.2)	78.4 (78.0-78.8)	0.876 (0.874-0.879)	0.898 (0.896-0.900)

a Model Demo: age, gender, BMI, and waist-hip ratio; Model CGM: Demo + CGM features; Model HbA1c: Demo + CGM + HbA1c.

b area under the receiver operating characteristic curve.

c area under the precision recall curve.

d CGM: continuous glucose monitoring.

e LR: logistic regression.

f SVM: support vector machine.

To further assess the feasibility of using CGM data to accurately identify individuals with prediabetes whose HbA_1c_ were in the normoglycemic range, we next focused on individuals with HbA_1c_ levels <5.7% (n=292) and compared the prediction outcomes for this subpopulation. We set the benchmark for comparison using the prediabetes cut-off defined by HbA_1c_ ≥5.7%, resulting in a specificity of 100%, sensitivity of 0%, and a misclassification rate of 25.7%. The ROC AUC and area under the precision recall curve values of the models were significantly higher than 0.5 and the test results yielded a slightly higher misclassification rate of 30%‐32%, a lower specificity of 73%‐80%, but a much higher sensitivity rate of 40%‐54% compared to the benchmark. SVM was slightly superior to LR in overall predictive performance and the omission of HbA_1c_ value as a variable resulted in a better model performance (Table 4).

**Table 4.** Prediction accuracy, including misclassification rates, specificity, and sensitivity, for those in the testing sets with glycated hemoglobin (HbA_1c_) <5.7%. This evaluation was conducted for model variants that utilized the 2-step prediction strategy and drew inference from all data points in the training sets, irrespective of their HbA_1c_ levels. The data are presented as the mean and 95% confidence intervals, derived from 1000 random splits into training and testing sets.

Model^a^	Misclassification (%)	Specificity (%)	Sensitivity (%)	ROC AUC^b^	PRC AUC^c^
CGM^d^, mean (95% CI)					
LR^e^	31.1 (30.8-31.5)	74.4 (74.1-74.8)	52.6 (51.9-53.3)	0.679 (0.674-0.683)	0.655 (0.650-0.659)
SVM^f^	32.0 (31.6-32.3)	72.7 (72.3-73.1)	54.4 (53.7-55.1)	0.703 (0.698-0.707)	0.640 (0.636-0.645)
HbA_1c_, mean (95% CI)					
LR	30.8 (30.5-31.1)	79.5 (79.1-79.8)	39.2 (38.5-39.9)	0.663 (0.659-0.668)	0.657 (0.652-0.661)
SVM	30.3 (30.0-30.6)	77.9 (77.5-78.2)	45.9 (45.2-46.6)	0.690 (0.686-0.695)	0.634 (0.629-0.638)
HbA_1c_ ≥5.7% cut-off (benchmark)	25.7	100	0	0	0

a Model Demo: age, gender, BMI, and waist-hip ratio; Model CGM: Demo + CGM features; Model HbA_1c_: Demo + CGM + HbA_1c_.

b area under the receiver operating characteristic curve.

c area under the precision recall curve.

d CGM: continuous glucose monitoring.

e LR: logistic regression.

f SVM: support vector machine.

## Discussion

Our findings demonstrate that combining CGM data together with HbA_1c_ greatly improved the sensitivity of detecting prediabetes. The best strategy was a 2-step approach where those with HbA_1c_ ≥5.7% were classified as having prediabetes, and the model was then used to detect those with prediabetes in the remaining population with HbA_1c_ <5.7% (who, otherwise, would have been classified as normoglycemic by HbA_1c_ alone). In this study, if HbA_1c_ alone was used to detect presence of prediabetes, of those who had HbA_1c_ <5.7%, 26% were prediabetic either by fasting and/or 2-hour plasma glucose, and would have been misclassified as normoglycemic. We feel that the compromise of losing specificity for a much higher sensitivity is a good strategy for prediabetes screening, as it will increase the chances of detecting those at risk of T2DM, while the false positives will still benefit from a diabetes prevention program that improves their lifestyle and diet choices. These findings can be implemented in a clinical setting, where general practitioners are able to improve HbA_1c_’s detection of prediabetes by adding the CGM into their arsenal of screening tools.

Our model to identify prediabetes had a sensitivity of 81.8% and specificity of 72.7%, a performance comparable to other CGM modeling studies. Acciaroli et al [14] reported an 86% sensitivity in identifying those with impaired glucose tolerance in a Caucasian population, while Kaufman et al [15] reported an 86% sensitivity and a specificity of 71%‐78% in identifying those with prediabetes from a study in India. Comparing the 2 classification algorithms employed in our study, SVM performed slightly better than LR, and the addition of HbA_1c_ as a variable greatly improved the CGM model. This was as expected since HbA_1c_ is, by definition, one of the parameters for prediabetes classification in the ADA criteria used in this study [18]. However, in the 2-step approach where those with HbA_1c_ ≥5.7% were automatically categorized as having prediabetes, further addition of HbA_1c_ as a variable was no longer beneficial to the CGM model. While we performed robust internal validation through repeated random subsampling to minimize overfitting, we acknowledge this does not substitute for external validation in independent cohorts. External validation would be needed to assess generalizability to other Asian populations and health care settings.

While the current consensus for time-in-range among patients with diabetes is 3.9‐10.0 mmol/L, there is an emerging secondary measure, termed time-in-tight-range of 3.9‐7.8 mmol/L, which is believed to better represent normoglycemia [22,23]. Nondiabetic participants of Western descent have an average time-in-tight-range of 96%‐97% [24,25]. However, in a Chinese population, the corresponding proportion was only 93% [26]. Moreover, it has been reported that nondiabetic individuals have a nonnegligible time spent in the hypoglycemic range of <3.9 mmol/L, calling to question whether the 3.9 mmol/L cut-off is relevant in persons without diabetes [24,26,27]. In our population, the amount of time spent in <3.9 mmol/L was 2.9% with the range almost exclusively in 3.0‐3.9 mmol/L. While we acknowledge that there have been reports of the Freestyle Libre CGM sensor underperforming in the hypoglycemic range [28], reducing the lower bound cut-off from 3.9 to 3.0 mmol/L supports the current recommendation of using 3.0 mmol/L to define clinically important hypoglycemia instead [22]. Hence, our recommended target time-in-range was 3.0‐7.8 mmol/L, which captured an average of 96.3% of time spent in this glucose range in an Asian nondiabetic population.

As the CGM provides real-time biofeedback to the user, monitoring with the CGM would serve a dual purpose of diagnosis and motivation for individuals to engage in healthy lifestyle behaviors, which in turn could improve glucose control. For prediabetic individuals, the use of CGM would be highly useful, as lifestyle modifications may be even more effective in reducing T2DM risk than metformin therapy (Diabetes Prevention Program) [29]. A short 10-day CGM along with exposure to activity and dietary insights, even without any specific dietary recommendations, was sufficient to significantly improve the time-in-range (3.0‐7.8 mmol/L) in nondiabetic individuals [30]. However, we acknowledge that we did not assess behavioral changes of the participants in this study and this claim would require prospective validation. In recent times, there is an increasing acceptability of CGM among the public even in the absence of diabetes [31], rating it useful to improving their lifestyle [27]. Furthermore, CGM may represent a more acceptable alternative for diabetes screening than the current gold-standard OGTT. In a gestational diabetes screening study, the pregnant participants perceived CGM as significantly more acceptable than the OGTT [32].

The main strengths of our study are the substantial sample size (for a clinical study) in which the major Asian groups of East, South, and Southeast Asia were represented, and the OGTT in addition with HbA_1c_ were performed to accurately determine the participant’s glycemic status. However, because the numbers of participants from other ethnicities such as Malay and Indian were low, we could not perform a subpopulation analysis to investigate whether the accuracy of the algorithm was affected by ethnicity. One limitation from our study design is that participants were not blinded to their own CGM data, which may have promoted healthier behaviors and improved their glucose profiles over the course of the recording period. Thus, the CGM profile may less accurately reflect the glycemic status from the OGTT and HbA_1c_ tests performed earlier (within 3 months). Our study population also did not include those with more severe hyperglycemia, that is, T2DM, hence it cannot provide a comprehensive discrimination between the varying degrees of glucose dysregulation and the possibility of detecting T2DM at the early stage of the disease. Lastly, while our study reported a prevalence of prediabetes at 47%, this is only 3%‐9% higher than other Asian studies with a Chinese-majority population, ranging from 38%‐44% [33,34]. For Yue et al [34] in particular, while their population demographics was approximately 10 years older than our study, their criteria for prediabetes only included impaired fasting glucose and HbA_1c_ 5.7%‐6.4%, while the impaired glucose tolerance was not taken into consideration; hence, the true prevalence for that study would have been higher than 44%. A single CGM assessment of 12 to 14 days in this cross-sectional study was sufficient to obtain adequate representation of the glucose variability that was distinguishable between the normoglycemia and prediabetes groups in this cohort. However, as CGM patterns may vary with seasonal changes, dietary patterns, or life circumstances, which a single assessment may not capture, future longitudinal work could examine whether repeated CGM assessments improve risk stratification or predict progression from prediabetes to T2DM.

In conclusion, the addition of CGM to HbA_1c_ in a 2-step approach, using either LR or SVM, greatly improved the sensitivity of detecting those at risk of T2DM in an Asian population. Given the benefits of the CGM to optimize lifestyle behaviors and its growing interest and acceptability among the nondiabetic population, CGM is an increasingly promising alternative to the classic OGTT for screening individuals at risk for T2DM in clinical practice.

## Supplementary material

10.2196/81520 Multimedia Appendix 1 Supplementary methods and results.

## Abbreviations

ADA: American Diabetes Association
APT-2D: Assessing the Progression to Type-2 Diabetes
CGM: continuous glucose monitoring
CGM-APT2D: Continuous Glucose Monitoring to Assess Glucose Dysregulation in Progression to Type-2 Diabetes
HbA_1c_: glycated hemoglobin
LR: logistic regression
OGTT: oral glucose tolerance test
ROC AUC: area under the receiver operating characteristic curve
SVM: support vector machine
T2DM: type 2 diabetes mellitus
