# Supplementary material for: Integration of Continuous Glucose Monitoring With HbA1c to Improve the Detection of Prediabetes in Asian Individuals: Model Development Study
Source: JMIR Diabetes. 2026 Apr 27;11:e81520. doi: 10.2196/81520 (PMC13118137; doi:10.2196/81520)
Supplement: Multimedia Appendix 1 [file diabetes-v11-e81520-s001.docx]

**SUPPLEMENTARY INFORMATION**

***Supplementary Methods***

**Derivation of the CGM summary statistics**

We first excluded potentially inaccurate CGM recordings, specifically those captured within the initial 24 hours, and merged records from different devices worn by the same individual when applicable. Let $\left\{ y_{i} \right\}_{1:n}$ represent the time series of records for an individual and $\left\{ t_{i} \right\}_{1:n}$ denote the corresponding time points for these records (in hours), with $n$ being the total number of valid observations. We computed 13 summary statistics for each individual, aiming at capturing centrality (mean) and spread (maximum, minimum, standard deviation, coefficient of variation [CV] and mean amplitude of glycemic excursion [MAGE]). We additionally included proportions of time spent within abnormal and normal glucose ranges (>7.8, between 3 and 7.8, and < 3 mmol/L), along with glucose excursion metrics (the average rise, fall, and their corresponding rates). A full list of the equations used to derive these metrics follows, but we excluded p(CGM>7.8) from subsequent prediction analyses due to collinearity.

1. Maximum (mmol/L) ${=max}_{\{i=1,...,n\}} y_{i}$
2. Minimum (mmol/L) ${=min}_{\{i=1,...,n\}} y_{i}$
3. Mean (mmol/L) $=\sum_{i=1}^{n} y_{i} /n:=\bar{y}$
4. Standard deviation (mmol/L) $=\sqrt{{\sum_{i=1}^{n} {(y}_{i}-\bar{y})}^{2}/(n-1)}:= sd(y)$
5. CV $=\bar{y}/sd(y)$
6. MAGE (mmol/L) $=\sum_{j=1}^{m} \left| y_{i}- \bar{y} \right|\cdot I\left( \left| y_{i}- \bar{y} \right|>\left. sd(y \right) \right)/m$ where $m=\sum_{i=1}^{n} I\left( \left| y_{i}- \bar{y} \right|>\left. sd(y \right) \right)$, and $I(\cdot)$ is the indicator function.
7. p(CGM>7.8) $=\sum_{i=1}^{n} I\left( y_{i}>7.8 \right)/n$
8. p(3<CGM<7.8) $=\sum_{i=1}^{n} I\left( {3<y}_{i}<7.8 \right)/n$
9. p(CGM<3) $=\sum_{i=1}^{n} I\left( y_{i}<3 \right) /n$
10. Rise (mmol/L) $=\sum_{k=1}^{K-1} ( P_{k+1}-V_{k})/(K-1)$ where $P_{k}$ and $V_{k}$ are values for peak and valley (to be elaborated in the following section).
11. Fall (mmol/L) $=\sum_{k=1}^{K} {(P}_{k} -V_{k})/K$
12. Rise rate (mmol/L/h) $=\sum_{k=1}^{K-1} \frac{P_{k+1}- V_{k}}{t_{P,k+1}-t_{V,k}}/(K-1)$
13. Fall rate (mmol/L/h) $=\sum_{k=1}^{K} \frac{P_{k}-V_{k}}{t_{P,k}-t_{V,k}}/K$

**Procedure to detect local extremes**

Let $P_{k}$ and $V_{k}$ respectively be the $k$-th local peak and valley in the time series of $\left\{ y_{i} \right\}_{1:n}$. We set the distances between consecutive peaks and valleys, $P_{k}-V_{k}$ and $P_{k+1}-V_{k}$, to be at least $D$ (set as 2mmol/L in our case). Following peakdet [1], we used the following procedure:

1. Initialize $i=1,k=1,X_{1}=\infty,X_{2}=-\infty,t_{1}=t_{2}=0$.
2. Let $X=y_{i}$
3. If $X>X_{2}$ then $X_{2}=X, t_{2}=t_{i}$.
4. If $X<X_{1}$ then $X_{1}=X, t_{1}=t_{i}$.
5. Search for peak $P_{k}$: if $X<X_{2}-D$ then set
   1. $P_{k}=X_{2},t_{P,k}=t_{2}$ and
   2. $X_{1}=X,t_{1}=t_{i}$.
6. Increment $i$ by one.
7. Repeat step 2) to 6) until $P_{k}$ is found
8. Repeat step 2) and 4)
9. Search for valley $V_{k}$: if $X>X_{1}+D$ then
   1. $V_{k}=X_{1}, t_{V,k}=t_{1}$
   2. $X_{2}=X, t_{2}=t_{i}$
10. Increment $i$ by one.
11. Repeat step 8) to 10) until $V_{k}$ is found
12. Increment $k$ by one.
13. Go back to step 2) until $i=n$.

**Predictor list for prediction models**

**Table S1.** List of predictors (row) in each of the three model variants (column).

| **_Predictors_  ^Model^** | **Demo** | **CGM** | **HbA1c** |
| --- | --- | --- | --- |
| **Age & gender** | ✓ | ✓ | ✓ |
| **BMI & waist-to-hip ratio** | ✓ | ✓ | ✓ |
| **12 CGM summary statistics** |  | ✓ | ✓ |
| **HbA1c value** |  |  | ✓ |

**Random subsampling validation to evaluate predictive efficacy**

Since the machine learning approach is liable to suffer from over-fitting in cases of small sample sizes [2], we adopted a random subsampling validation approach to assess the prediction accuracy. For each model variant, we conducted 1000 random splits of the complete dataset into training and testing sets, with each testing set comprising 100 samples. We performed inference on the training sets, predicted the PD status on the corresponding testing sets, and compared these predictions with the observations. Subsequently, we computed the misclassification, specificity, and sensitivity rates, as well as area under the curve (AUC) using the aggregated test datasets and derived the corresponding 95% confidence intervals using bootstrapping. Table 2 in the main manuscript and Supplemental Table S6 utilized this approach.

The two-step prediction approach distinguishes individuals based on whether their HbA1c is above or below 5.7%. For those with HbA1c levels of ≥ 5.7%, they are automatically categorized as PD bypassing the predictive model since they have already met the PD threshold, while the model is trained exclusively on individuals with HbA1c < 5.7%. The detailed process involves the following steps:

1. The data is randomly split into training and testing sets;
2. The model is trained using either logistic regression or support vector machine, on the full training dataset irrespective of their HbA1c levels;
3. The model fits are utilized to predict the prediabetic risks and status of those in the testing set with HbA1c level lower than 5.7%, while those in the testing test with HbA1c higher than 5.6% are automatically predicted as prediabetic;
4. Comparisons are made between predictions and observations of all individuals in the testing set;
5. Repeat 1)­–4) 1000 times to obtain robust estimates for prediction accuracy.

This method guarantees a comprehensive evaluation of the models’ performance across varying training and testing scenarios, enhancing the relevance of the results to real-world applications. Tables 3 and 4 in the main manuscript and Supplemental Table S7 utilized this approach.

**Sensitivity analysis on the threshold of 5.6% in the two-step approach.**

We employed alternative thresholds, ranging from 5.6% to 6.3%, to identify individuals who are ‘certain’ to be PD (i.e., those without the need for machine learning algorithms to predict their PD risk) in the two-step approach. Specifically, we categorized individuals with HbA1c levels higher than the selected threshold (denoted as $s$) as PD and then trained the prediction algorithms on people in the training sets with HbA1c levels not exceeding $s$. Predictions were then made on individuals in the testing sets with HbA1c levels not exceeding $s$. Finally, we compared the predicted results with the observations for all individuals in the testing sets, and computed the summary statistics for prediction accuracy over the 1000 randomly selected testing sets. Supplemental Tables S2 and S3 shows the results of the sensitivity analysis using the logistic regression and support vector machine algorithms respectively.

***Supplementary Results***


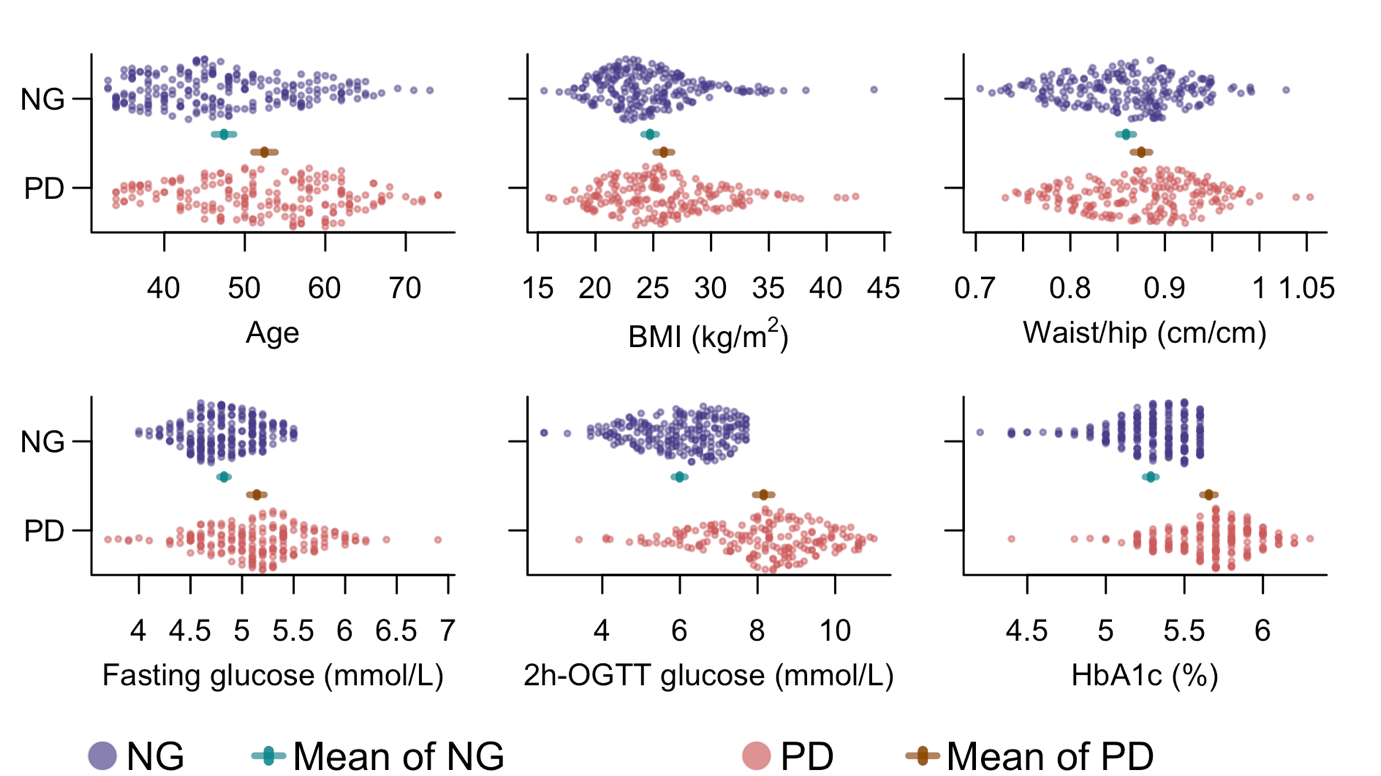


**Figure S1.** Demographic and clinical glucose measurements in Asians without diabetes.

**Table S2.** Sensitivity analysis using logistic regression algorithm. This evaluation is conducted for model variants which utilized the two-step prediction strategy and drew inference exclusively from those in the training sets with HbA1c level no higher than a threshold ranging from 5.6% to 6.3%.

| **Model** | **HbA1c % cut-off** | **Misclassification (%)** | **Specificity (%)** | **Sensitivity (%)** | **ROC AUC** | **PRC AUC** |
| --- | --- | --- | --- | --- | --- | --- |
| CGM | 5.6 | 22.5 (22.2,22.7) | 74.4 (74.1,74.8) | 81.1 (80.7,81.4) | 0.872 (0.869,0.874) | 0.893 (0.891,0.895) |
|  | 5.7 | 22.5 (22.2,22.7) | 74.4 (74.1,74.8) | 81.1 (80.7,81.4) | 0.872 (0.869,0.874) | 0.893 (0.891,0.895) |
|  | 5.8 | 28.7 (28.5,29) | 74.4 (74.1,74.8) | 67.6 (67.1,68) | 0.778 (0.775,0.781) | 0.784 (0.781,0.788) |
|  | 5.9 | 28.7 (28.5,29) | 74.4 (74.1,74.8) | 67.6 (67.1,68) | 0.778 (0.775,0.781) | 0.784 (0.781,0.788) |
|  | 6.0 | 32.1 (31.8,32.4) | 74.4 (74.1,74.8) | 60.3 (59.9,60.8) | 0.729 (0.726,0.732) | 0.702 (0.697,0.706) |
|  | 6.1 | 32.2 (31.9,32.4) | 74.4 (74.1,74.8) | 60.2 (59.7,60.6) | 0.725 (0.722,0.728) | 0.682 (0.677,0.686) |
|  | 6.2 | 32.2 (31.9,32.4) | 74.4 (74.1,74.8) | 60.2 (59.7,60.6) | 0.725 (0.722,0.728) | 0.682 (0.677,0.686) |
|  | 6.3 | 32.3 (32,32.6) | 74.4 (74.1,74.8) | 59.9 (59.5,60.3) | 0.723 (0.719,0.726) | 0.663 (0.658,0.667) |
| HbA1c | 5.6 | 22.3 (22,22.5) | 79.5 (79.1,79.8) | 75.7 (75.3,76.1) | 0.866 (0.863,0.868) | 0.889 (0.887,0.891) |
|  | 5.7 | 22.3 (22,22.5) | 79.5 (79.1,79.8) | 75.7 (75.3,76.1) | 0.866 (0.863,0.868) | 0.889 (0.887,0.891) |
|  | 5.8 | 23.2 (22.9,23.5) | 79.5 (79.1,79.8) | 73.7 (73.3,74) | 0.842 (0.84,0.845) | 0.852 (0.849,0.854) |
|  | 5.9 | 23.2 (22.9,23.5) | 79.5 (79.1,79.8) | 73.7 (73.3,74) | 0.842 (0.84,0.845) | 0.852 (0.849,0.854) |
|  | 6.0 | 23.2 (23,23.5) | 79.5 (79.1,79.8) | 73.6 (73.2,74) | 0.839 (0.836,0.841) | 0.837 (0.834,0.84) |
|  | 6.1 | 23.2 (23,23.5) | 79.5 (79.1,79.8) | 73.6 (73.2,74) | 0.838 (0.836,0.841) | 0.834 (0.831,0.837) |
|  | 6.2 | 23.2 (23,23.5) | 79.5 (79.1,79.8) | 73.6 (73.2,74) | 0.838 (0.836,0.841) | 0.834 (0.831,0.837) |
|  | 6.3 | 23.2 (23,23.5) | 79.5 (79.1,79.8) | 73.6 (73.2,74) | 0.838 (0.836,0.841) | 0.829 (0.826,0.833) |

The data are presented as the mean and 95% confidence intervals, derived from 1000 random splits into training and testing sets. Model Demo: age, gender, BMI and waist-hip ratio; Model CGM: Demo + CGM features; Model HbA1c: Demo + CGM + HbA1c.

**Table S3.** Sensitivity analysis using support vector machine algorithm. This evaluation is conducted for model variants which utilized the two-step prediction strategy and drew inference exclusively from those in the training sets with HbA1c level no higher than a threshold ranging from 5.6% to 6.3%.

| **Model** | **HbA1c % cut-off** | **Misclassification (%)** | **Specificity (%)** | **Sensitivity (%)** | **ROC AUC** | **PRC AUC** |
| --- | --- | --- | --- | --- | --- | --- |
| CGM | 5.6 | 23.1 (22.8,23.4) | 72.7 (72.3,73.1) | 81.8 (81.4,82.2) | 0.881 (0.879,0.883) | 0.899 (0.897,0.901) |
|  | 5.7 | 23.1 (22.8,23.4) | 72.7 (72.3,73.1) | 81.8 (81.4,82.2) | 0.881 (0.879,0.883) | 0.899 (0.897,0.901) |
|  | 5.8 | 28.3 (28,28.6) | 72.7 (72.3,73.1) | 70.6 (70.1,71) | 0.795 (0.792,0.798) | 0.798 (0.795,0.801) |
|  | 5.9 | 28.3 (28,28.6) | 72.7 (72.3,73.1) | 70.6 (70.1,71) | 0.795 (0.792,0.798) | 0.798 (0.795,0.801) |
|  | 6.0 | 31.5 (31.2,31.8) | 72.7 (72.3,73.1) | 63.7 (63.2,64.1) | 0.747 (0.744,0.75) | 0.721 (0.717,0.725) |
|  | 6.1 | 31.7 (31.5,32) | 72.7 (72.3,73.1) | 63.1 (62.7,63.5) | 0.742 (0.739,0.745) | 0.706 (0.701,0.71) |
|  | 6.2 | 31.7 (31.5,32) | 72.7 (72.3,73.1) | 63.1 (62.7,63.5) | 0.742 (0.739,0.745) | 0.706 (0.701,0.71) |
|  | 6.3 | 31.9 (31.6,32.2) | 72.7 (72.3,73.1) | 62.8 (62.4,63.3) | 0.740 (0.736,0.743) | 0.693 (0.689,0.698) |
| HbA1c | 5.6 | 21.9 (21.6,22.1) | 77.9 (77.5,78.2) | 78.4 (78,78.8) | 0.876 (0.874,0.879) | 0.898 (0.896,0.9) |
|  | 5.7 | 21.9 (21.6,22.1) | 77.9 (77.5,78.2) | 78.4 (78,78.8) | 0.876 (0.874,0.879) | 0.898 (0.896,0.9) |
|  | 5.8 | 24.5 (24.2,24.7) | 77.9 (77.5,78.2) | 72.8 (72.4,73.2) | 0.840 (0.837,0.842) | 0.849 (0.846,0.851) |
|  | 5.9 | 24.5 (24.2,24.7) | 77.9 (77.5,78.2) | 72.8 (72.4,73.2) | 0.840 (0.837,0.842) | 0.849 (0.846,0.851) |
|  | 6.0 | 25.2 (24.9,25.4) | 77.9 (77.5,78.2) | 71.3 (70.9,71.7) | 0.829 (0.827,0.832) | 0.829 (0.826,0.832) |
|  | 6.1 | 25.2 (24.9,25.4) | 77.9 (77.5,78.2) | 71.3 (70.9,71.7) | 0.829 (0.827,0.832) | 0.829 (0.826,0.832) |
|  | 6.2 | 25.2 (24.9,25.4) | 77.9 (77.5,78.2) | 71.3 (70.9,71.7) | 0.829 (0.827,0.832) | 0.829 (0.826,0.832) |
|  | 6.3 | 25.2 (24.9,25.4) | 77.9 (77.5,78.2) | 71.3 (70.9,71.7) | 0.829 (0.826,0.831) | 0.828 (0.825,0.831) |

The data are presented as the mean and 95% confidence intervals, derived from 1000 random splits into training and testing sets. Model Demo: age, gender, BMI and waist-hip ratio; Model CGM: Demo + CGM features; Model HbA1c: Demo + CGM + HbA1c.

**Table S4.** CGM glucose metrics in Asians without diabetes.

| **Variable** | **Total (N=406)** | **Normoglycemia**  **(NG) (N=217)** | **Prediabetes**  **(PD) (N=189)** | **P-value** |
| --- | --- | --- | --- | --- |
| **Maximum (mmol/L)** | 9.6 (8.7, 10.8) | 9.1 (8.3, 10.2) | 10.2 (9.1, 11.5) | <0.001 |
| **Minimum (mmol/L)** | 3.1 (2.5, 3.6) | 3.1 (2.5, 3.6) | 3.2 (2.4, 3.7) | 0.283 |
| **Mean (mmol/L)** | 5.2 (4.8, 5.6) | 5.0 (4.7, 5.4) | 5.4 (4.9, 5.8) | <0.001 |
| **SD (mmol/L)** | 1.0 (0.9, 1.2) | 1.0 (0.8, 1.1) | 1.10 (0.9, 1.3) | <0.001 |
| **CV (SD/mean) (%)** | 19.8 (17.1, 23.7) | 19.2 (16.5, 22.2) | 20.7 (17.9, 24.4) | 0.002 |
| **MAGE (mmol/L)** | 5.9 (5.4, 6.5) | 5.6 (5.2, 6.3) | 6.2 (5.7, 6.9) | <0.001 |
| **p(CGM>7.8 mmol/L) (%)** | 2.2 (0.6, 5.0) | 1.3 (0.3, 3.6) | 3.5 (1.4, 8.4) | <0.001 |
| **p(3.0<CGM<7.8 mmol/L) (%)** | 96.3 (93.1, 98.5) | 97.0 (94.9, 98.7) | 95.5 (90.0, 97.9) | <0.001 |
| **p(CGM<3.0 mmol/L) (%)** | 0.2 (0.0, 1.4) | 0.3 (0.0, 1.9) | 0.1 (0.0, 0.9) | 0.006 |
| **Rise (mmol/L)** | 3.5 (3.2, 3.9) | 3.4 (3.1, 3.7) | 3.6 (3.3, 4.0) | <0.001 |
| **Fall (mmol/L)** | -3.4 (-3.9, -3.1) | -3.3 (-3.6, -3.0) | -3.6 (-4.1, -3.2) | <0.001 |
| **Rise rate (mmol/L)** | 1.5 (1.2, 1.8) | 1.4 (1.1, 1.8) | 1.5 (1.2, 1.9) | 0.039 |
| **Fall rate (mmol/L)** | -1.3 (-1.7, -1.0) | -1.3 (-1.6, -1.0) | -1.4 (-1.8, -1.1) | 0.005 |

Data are displayed as median (IQR). Comparison between normoglycemia (NG) and prediabetes (PD) groups was performed using Mann-Whitney U test.

**Table S5.** CGM glucose metrics in Asians without diabetes, utilizing the conventional cut-off for time-in-range (3.9-10 mmol/L).

| **Variable** | **Total (N=406)** | **Normoglycemia**  **(NG) (N=217)** | **Prediabetes**  **(PD) (N=189)** | **P-value** |
| --- | --- | --- | --- | --- |
| **p(CGM>13.9 mmol/L) (%)** | 0.0 (0.0, 0.0) | 0.0 (0.0, 0.0) | 0.0 (0.0, 0.0) | 0.10 |
| **p(CGM>10 mmol/L) (%)** | 0.0 (0.0, 0.4) | 0.0 (0.0, 0.1) | 0.1 (0.0, 0.7) | <0.001 |
| **p(3.9<CGM<10 mmol/L) (%)** | 96.0 (87.0, 99.0) | 95.0 (82.0, 99.0) | 97.0 (90.0, 99.0) | 0.02 |
| **p(CGM<3.9 mmol/L) (%)** | 2.9 (0.4, 13.0) | 4.9 (0.7, 18.0) | 1.6 (0.3, 7.1) | <0.001 |
| **p(CGM<3.0 mmol/L) (%)** | 0.2 (0.0, 1.3) | 0.3 (0.0, 1.9) | 0.1 (0.0, 0.8) | 0.006 |

Data are displayed as median (IQR). Comparisons between normoglycemia (NG) and prediabetes (PD) groups were performed using Mann-Whitney U test.

**Table S6.** Prediction accuracy of PD, in terms of misclassification rates, specificity, and sensitivity, for random forest (RF) and XGBoost (XGB) models using distinct predictor sets and classification algorithms.

| **Model** | **Misclassification (%)** | **Specificity (%)** | **Sensitivity (%)** | **ROC AUC** |
| --- | --- | --- | --- | --- |
| **Demo (RF)** | 41.9 (41.6,42.2) | 63.8 (63.4,64.2) | 51.4 (50.9,51.8) | 0.602 (0.598,0.605) |
| **Demo (XGB)** | 40.6 (40.3,40.9) | 74.7 (74.3,75.0) | 41.7 (41.2,42.1) | 0.621 (0.617,0.624) |
| **CGM (RF)** | 33.7 (33.4,34.0) | 72.3 (71.9,72.7) | 59.3 (58.8,59.7) | 0.716 (0.713,0.719) |
| **CGM (XGB)** | 33.2 (32.9,33.5) | 73.3 (72.9,73.6) | 59.2 (58.7,59.6) | 0.721 (0.718,0.725) |
| **HbA1c (RF)** | 18.8 (18.6,19.0) | 90.2 (89.9,90.4) | 70.7 (70.3,71.2) | 0.862 (0.859,0.864) |
| **HbA1c (XGB)** | 18.9 (18.6,19.1) | 90.8 (90.6,91.1) | 69.8 (69.3,70.2) | 0.864 (0.862,0.866) |

The data are presented as the mean and 95% confidence intervals, derived from 1000 random splits into training and testing sets. RF and XGB abbreviations refer to random forest and XGBoost, respectively. Model Demo: age, gender, BMI and waist-hip ratio; Model CGM: Demo + CGM features; Model HbA1c: Demo + CGM + HbA1c.

**Table S7.** Prediction accuracy of PD, including misclassification rate, specificity, and sensitivity, for all data points within the testing sets using random forest (RF) and XGBoost (XGB) models. This evaluation was conducted for model variants which utilized the two-step prediction strategy and drew inference from all data points in the training sets, irrespective of their HbA1c levels.

| **Model** | **Misclassification (%)** | **Specificity (%)** | **Sensitivity (%)** | **ROC AUC** | **PRC AUC** |
| --- | --- | --- | --- | --- | --- |
| **CGM (RF)** | 23.9 (23.6,24.1) | 72.3 (71.9,72.7) | 80.6 (80.2,80.9) | 0.869 (0.867,0.871) | 0.892 (0.890,0.894) |
| **CGM (XGB)** | 23.3 (23.1,23.6) | 73.3 (72.9,73.6) | 80.6 (80.2,80.9) | 0.872 (0.870,0.875) | 0.894 (0.892,0.896) |
| **HbA1c (RF)** | 18.8 (18.6,19.0) | 90.2 (89.9,90.4) | 70.7 (70.3,71.2) | 0.862 (0.859,0.864) | 0.889 (0.887,0.891) |
| **HbA1c (XGB)** | 18.9 (18.6,19.1) | 90.8 (90.6,91.1) | 69.8 (69.3,70.2) | 0.864 (0.862,0.866) | 0.889 (0.887,0.891) |

The data are presented as the mean and 95% confidence intervals, derived from 1000 random splits into training and testing sets. RF and XGB abbreviations refer to random forest and XGBoost, respectively. ROC and PRC refer to receiver operating characteristic and precision recall curve respectively. Model Demo: age, gender, BMI and waist-hip ratio; Model CGM: Demo + CGM features; Model HbA1c: Demo + CGM + HbA1c.

**References**

[1] Eli Billauer. peakdet: Peak detection using MATLAB (non-derivative local extremum, maximum, minimum) n.d. https://billauer.co.il/blog/2009/01/peakdet-matlab-octave/ (accessed January 11, 2024).

[2] Vabalas A, Gowen E, Poliakoff E, Casson AJ. Machine learning algorithm validation with a limited sample size. PLOS ONE 2019;14:e0224365. https://doi.org/10.1371/journal.pone.0224365.
